# Plasma haem oxygenase-1 and interleukin-6 as adjunct host biomarkers associated with malaria

**DOI:** 10.3389/fimmu.2026.1835939

**Published:** 2026-05-15

**Authors:** Theophilus Wakai, Irrinus Kintung, Temitayo Ogundimu, Shalom Chinedu, Israel Afolabi

**Affiliations:** 1 Covenant Applied Informatics and Communication–Africa Centre of Excellence (CApIC-ACE), Ota, Nigeria; 2 Department of Biochemistry, College of Science and Technology, Covenant University, Ota, Nigeria; 3 Department of Computer and Information Sciences, College of Science and Technology, Covenant University, Ota, Nigeria; 4 Covenant University Public Health and Wellbeing Research Cluster (CUPHWERC), Covenant University, Ota, Nigeria

**Keywords:** biomarkers, haem oxygenase-1, inflammation, interleukin-6, malaria, oxidative stress, parasite density, thrombocytopenia

## Abstract

**Introduction:**

Malaria pathogenesis involves both parasite burden and host inflammatory and oxidative stress responses that contribute to haematological alterations. Full blood count (FBC)-derived indices have been explored as accessible surrogate biomarkers, reflecting systemic inflammatory changes; however, they provide limited mechanistic insight into underlying haem-driven oxidative stress pathways. Haem oxygenase-1 (HO-1) reflects cellular responses to free haem, while interleukin-6 (IL-6) mediates systemic inflammation. However, their combined role in relation to parasitaemia and haematological alterations in endemic populations remains insufficiently defined. This study evaluated plasma HO-1 and IL-6 as complementary host-response biomarkers in a hospital-based malaria-endemic population.

**Methods:**

This cross-sectional study was conducted at Covenant University Medical Centre (CUMC), Canaanland, Ota, Ogun State, Nigeria. A total of 647 individuals were screened, and 304 participants with complete microscopy and haematological data were retained in the analytical cohort (152 microscopy-positive and 152 microscopy-negative). An integrated biomarker subset of 40 participants with matched plasma samples and complete laboratory data was used for ELISA, correlation, and receiver operating characteristic (ROC) analyses. Group comparisons used Mann-Whitney U and Kruskal-Wallis tests, categorical variables were compared using chi-square or Fisher exact tests, and associations were assessed using Spearman correlation.

**Results:**

In the analytical cohort, microscopy-positive participants had lower haemoglobin, haematocrit, red-cell count, mean cell indices, and platelet counts, together with higher temperature and white-cell counts than microscopy-negative participants (all p< 0.001 for the major contrasts). Fever and anaemia were also substantially more frequent in microscopy-positive participants. In the biomarker subset, IL-6 was significantly higher in malaria-positive participants than malaria-negative participants (median 141.28 vs 106.58 pg/mL; p = 0.027), whereas HO-1 showed a non-significant upward shift (230.98 vs 193.73 ng/mL; p = 0.131). IL-6 correlated positively with HO-1 (rho = 0.702, p< 0.001), parasitaemia (rho = 0.387, p = 0.015), and temperature (rho = 0.432, p = 0.006), and negatively with haemoglobin (rho = -0.417, p = 0.008). ROC analysis showed moderate discriminatory performance for IL-6 and lower performance for HO-1. IL-6 yielded an AUC of 0.709 (95% CI 0.531–0.874), with sensitivity of 72.7% and specificity of 76.5% at the Youden-optimal threshold. HO-1 yielded an AUC of 0.641 (95% CI 0.440–0.826), while the combined IL-6 + HO-1 model yielded an AUC of 0.687 (95% CI 0.461–0.886).

**Conclusions:**

IL-6 showed better discriminatory performance than HO-1 in this cohort, while HO-1 demonstrated only modest and non-significant differences between groups. These findings suggest that IL-6 may be a more informative adjunct inflammatory marker in malaria, whereas the role of HO-1 requires further validation in larger studies.

## 1 Introduction

Malaria remains one of the most consequential infectious diseases worldwide, with the highest burden concentrated in sub-Saharan Africa. The 2025 World Malaria Report states that malaria continues to impose major morbidity and mortality, with Nigeria remaining among the countries contributing the largest share of global disease burden (1, 2). Yet parasite detection alone does not fully explain why some infected individuals experience greater physiological derangement than others (3–5).

Contemporary understanding of malaria pathogenesis has emphasised that severe or complicated disease arises from an interplay among parasite biomass, haemolysis, endothelial activation, coagulation disturbances, oxidative stress, and dysregulated inflammatory signalling (6–8). During blood-stage infection, parasitised erythrocytes rupture, releasing cell-free haemoglobin and free haem. Free haem is a potent pro-oxidant that can damage endothelium, amplify inflammatory cascades, and worsen tissue injury (9, 10). In this context, host-response biomarkers provide a mechanistic window into disease biology that is not captured by microscopy or rapid diagnostic testing alone (11, 12).

Haem oxygenase-1 (HO-1), encoded by *HMOX1* is a stress-responsive enzyme that catalyses haem degradation to biliverdin, carbon monoxide, and free iron. In malaria, HO-1 has been interpreted as a double-edged pathway: experimental and translational studies are consistent with protective effects through detoxification of free haem and mitigation of oxidative injury, while human studies have also linked heightened HO-1 expression with severe disease states in specific clinical settings (13, 14). This duality makes HO-1 biologically useful as both a mechanistic readout and a candidate adjunct biomarker (14, 15).

IL-6 is a multifunctional inflammatory cytokine involved in fever generation, hepatic acute-phase signalling, lymphocyte activation, endothelial perturbation, and the orchestration of innate and adaptive responses (16, 17). Earlier clinical studies demonstrated that circulating IL-6 is elevated in complicated malaria (18, 19), while newer systematic reviews and meta-analyses support an overall association between higher IL-6 concentrations and malaria-associated physiological disturbance (20, 21).

Despite the expanding literature on host biomarkers in malaria, several important gaps remain. First, many studies have investigated either inflammatory mediators or oxidative-stress pathways independently rather than examining both biological axes within an integrated host-response framework. Second, a large proportion of biomarker studies have been conducted in narrowly defined populations such as paediatric severe malaria cohorts (22) or pregnancy-associated malaria (23, 24), where host responses may differ substantially from those observed in general endemic populations.

Other investigations have focused on specific clinical contexts, including studies evaluating inflammatory cytokines as indicators of severe malaria in hospitalised paediatric populations. For example, Obeng-Aboagye et al. examined inflammatory cytokines as potential biomarkers for early diagnosis of severe malaria in children (25), while studies by Oluboyo et al. and Varo et al. investigated endothelial activation markers such as angiopoietins in relation to severe malaria pathophysiology and clinical outcomes (6, 26). In addition, Kortz et al. compared host biomarker profiles in malaria and non-malarial sepsis, highlighting differences in inflammatory and endothelial responses across febrile illnesses (27).

Generally, only a limited number of studies have evaluated the diagnostic or discriminatory performance of host biomarkers. For instance, Foko et al. identified CRP, Ang-2, Ang-2/1 ratio, PfHRP2, and platelet count as high-value biomarkers for predicting severe malaria and cerebral malaria (21). Systematic and meta-analytic assessments of inflammatory cytokines propose them as potential biomarkers for malaria disease (16, 20). This limitation is particularly relevant in endemic outpatient and mixed-population settings, where host biomarker signals may differ from those observed in highly selected severe-malaria cohorts. To the best of our knowledge, this study represents one of the earliest investigations to simultaneously evaluate plasma haem oxygenase-1 (HO-1) and interleukin-6 (IL-6) and assess their joint diagnostic performance in a general malaria-exposed population. Herein, we investigate plasma HO-1 and IL-6 levels in a hospital-based malaria-endemic population in southwestern Nigeria. Malaria-associated physiological disturbance was assessed using parasite density, haemoglobin concentration, platelet count, and fever. The study aimed to determine (i) whether plasma HO-1 and IL-6 are elevated in malaria infection, (ii) whether these biomarkers are associated with parasite-density and haematological indicators of malaria-associated physiological disturbance, and (iii) whether their combined diagnostic performance supports their potential utility as adjunct host biomarkers in endemic clinical settings.

## 2 Materials and methods

### 2.1 Study design and setting

**Figure 1:**
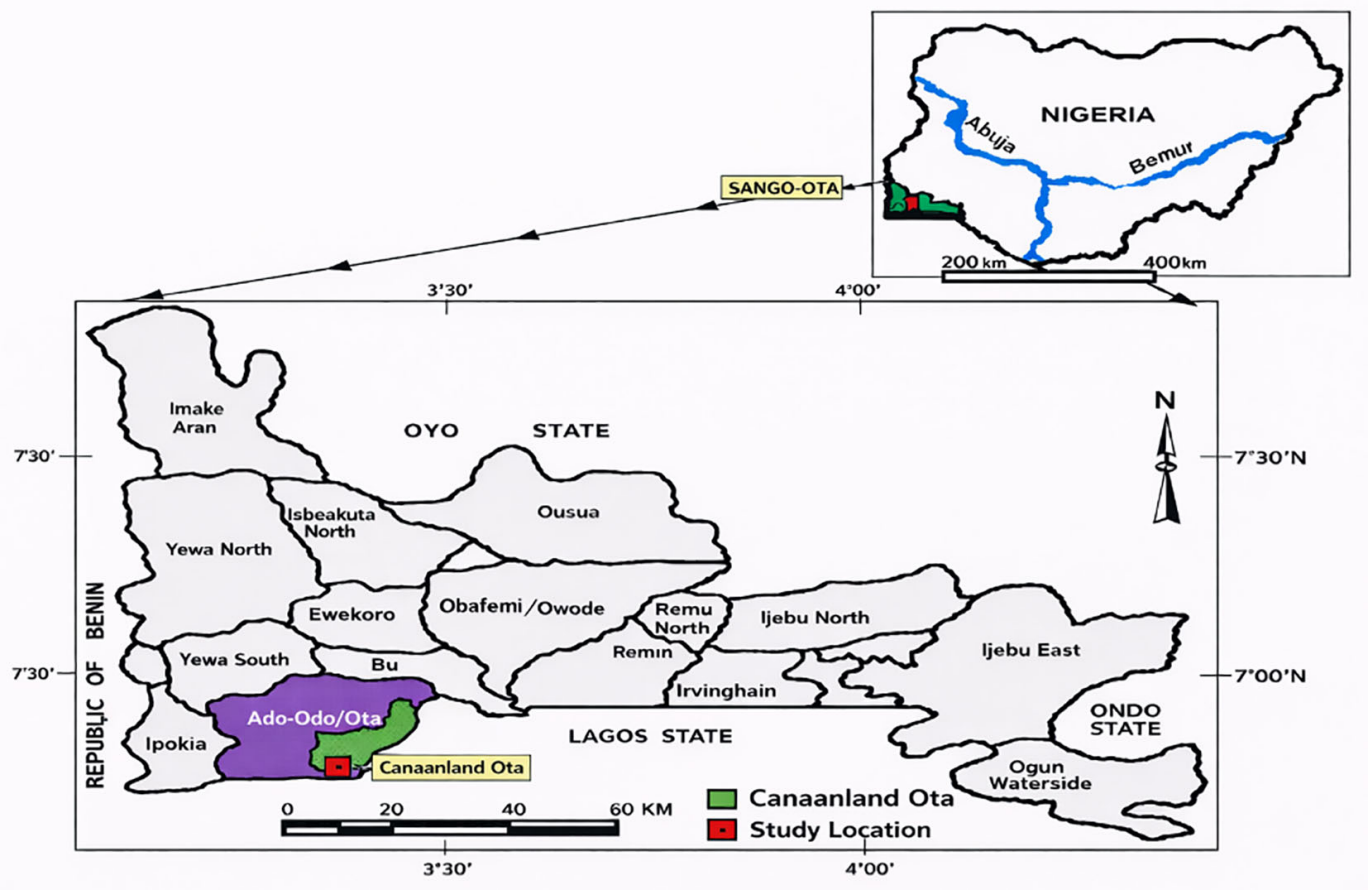
Map of the study area. Adapted from Akinbile et al. (28), under CC BY-NC-ND 4.0.

This study was conducted among residents of Canaanland and its adjoining communities in Ota, Ogun State, Nigeria (**Figure 1**). Canaanland is a prominent religious and educational enclave located in Ado-Odo/Ota Local Government Area, approximately 40 km from Lagos, Nigeria’s commercial capital. The area hosts the international headquarters of Living Faith Church Worldwide (Winners’ Chapel), Covenant University, Faith Academy Secondary School, and Kingdom Heritage Nursery and Primary School, forming a large residential and academic community with an estimated population of approximately 11, 000 residents, including church officials, academic and support staff, and students.

Geographically, Canaanland lies between latitudes 6°40′–6°42′N and longitudes 3°08′–3°10′E. The region experiences a tropical wet-and-dry climate, with a prolonged rainy season from April to October and a shorter dry season from November to March. These climatic conditions favour the breeding of Anopheles mosquito vectors, contributing to stable malaria transmission in the region (29).

The study was conducted between March and December 2025, spanning the onset and progression of the rainy season in southern Nigeria, a period often associated with increased malaria transmission due to enhanced mosquito breeding conditions (29, 30). Recent studies conducted among residents of Canaanland and surrounding communities reported a 44% symptomatic malaria prevalence (31), significant knowledge gaps and misconceptions about malaria causes, alongside suboptimal preventive practices and reliance on self-diagnosis (32). These characteristics make the facility an appropriate setting for investigating malaria infection and host biomarker responses in a mixed endemic population (31, 33).

### 2.2 Study population

Participant recruitment was conducted among individuals presenting for malaria screening or routine clinical evaluation at the Medical Centre and during community health outreaches within Canaanland.

Participants were recruited from two sources: (i) individuals presenting for clinical evaluation at the health facility, and (ii) participants enrolled through community outreach screening. Individuals presenting at the health facility with fever or symptoms suggestive of malaria, or with an axillary temperature ≥ 37.5 °C, were considered clinically eligible (34, 35). In contrast, outreach participants were enrolled irrespective of fever status to capture asymptomatic, subclinical, or malaria-negative individuals within the endemic population. All participants provided informed consent (or guardian consent for minors) prior to inclusion.

A total of 647 individuals meeting these initial eligibility criteria were screened for malaria. Participants who were currently receiving antimalarial therapy or had taken antimalarial medication within the preceding three weeks were excluded to minimise potential effects on parasite detection and biomarker measurements. In addition, individuals with microscopy-confirmed mixed *Plasmodium* infections were excluded to ensure consistency in the interpretation of malaria-associated host responses.

**Figure 2:**
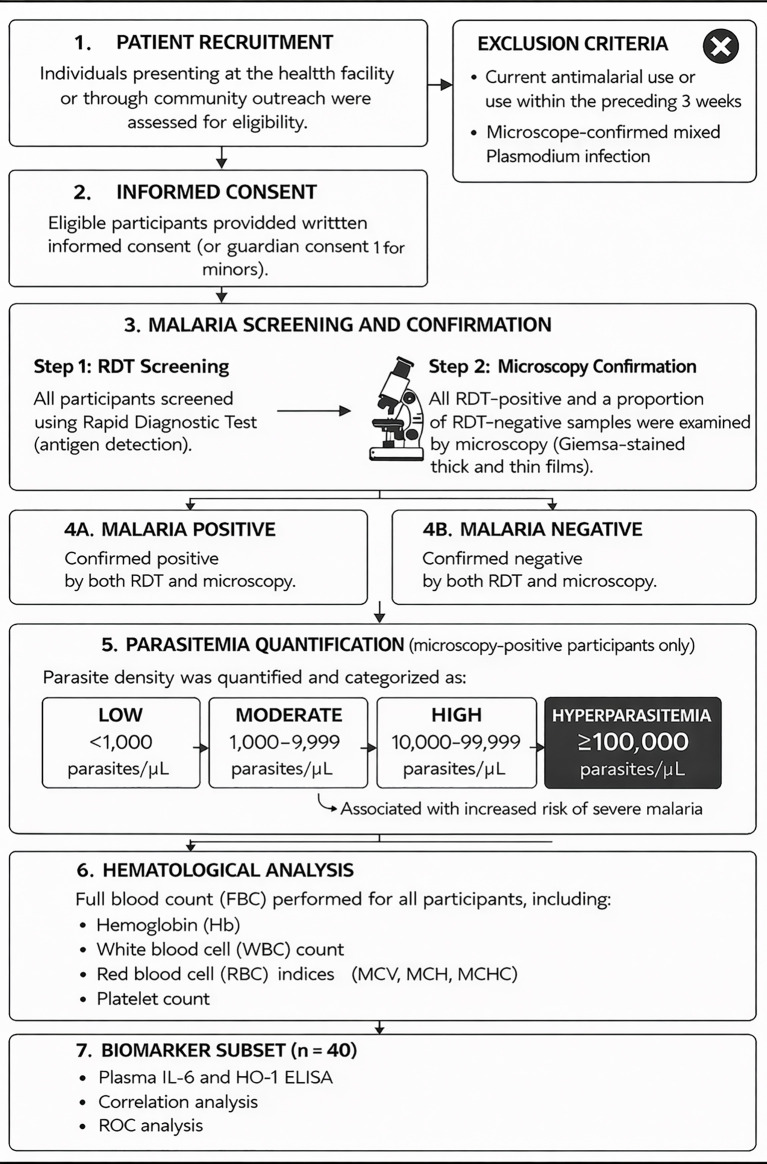
Study flow diagram.

An integrated biomarker subset of 40 participants was defined using a complete-case approach, including only individuals with fully matched plasma IL-6 and HO-1 measurements, parasitaemia data, and haematological profiles. Demographic variables, including age and sex, were retained (36). The study participant flow diagram illustrating recruitment, screening, laboratory assessment, and biomarker subset selection is summarized in **Figure 2**.

### 2.3 Sample collection and processing

Approximately 5 mL of venous blood was collected aseptically from each participant by venipuncture into ethylenediaminetetraacetic acid (EDTA)-anticoagulated tubes, following standard phlebotomy procedures. EDTA-anticoagulated blood was used for malaria microscopy and full blood count analysis. For plasma preparation, blood was collected in lithium-heparin tubes and was centrifuged at 3000 rpm for 10 minutes, separated and then aliquoted into sterile cryovials before being stored at −20 °C until biomarker analysis.

### 2.4 Malaria diagnosis and parasite quantification

Malaria screening was initially performed using the CareStart Malaria HRP2/pLDH (Pf/PAN) Combo rapid diagnostic test (Access Bio, Somerset, NJ, USA). Microscopic examination, considered the gold standard for malaria diagnosis, was used to define malaria status for the analytical comparisons in this manuscript. Thick and thin blood films were prepared on clean microscope slides using standard laboratory techniques (37, 38). The films were stained with 10% Giemsa solution and examined under a light microscope with a ×100 oil-immersion objective lens. Thick films were used for parasite detection, while thin films were used for species identification based on morphological characteristics, as described by Cheesbrough (39). Parasite density was quantified microscopically, and microscopy-positive biomarker cases were stratified as low, moderate, high, or hyperparasitaemia for malaria-associated physiological disturbance-related analyses (40, 41).

### 2.5 Haematological measurements

Full blood counts were performed using the Sysmex XS-1000i automated haematology analyser (Sysmex Corporation, Kobe, Japan). Parameters considered in the present work included haemoglobin concentration, red blood cell count, white blood cell count, and platelet count. Fever was recorded clinically and considered alongside parasite density, haemoglobin, and platelet count as a malaria-associated physiological disturbance-related variable (37).

### 2.6 Definition of parasite burden and clinical indicators

Malaria burden in this study was assessed using parasite density together with selected haematological and clinical indicators, including haemoglobin concentration, platelet count, and fever. These parameters are widely recognised as important clinical correlations of malaria-associated haemolysis, thrombocytopenia, and disease progression in endemic settings (21, 42).

#### 2.6.1 Parasite density estimation

Parasite density was determined from Giemsa-stained thick blood smears by counting the number of malaria parasites relative to 200 white blood cells (WBCs) under oil immersion microscopy. An assumed average leukocyte count of 8000 WBCs/µL was used for the calculation. Parasite density per microlitre of blood was estimated using the formula:

$\text{Parasites}/\mu \text{L}=\frac{\text{Number of parasites counted}\times 8000}{\text{Number of WBCs counted}}$For analytical purposes, parasite density was categorized as low (<1, 000 parasites/µL), moderate (1, 000–9, 999 parasites/µL), high (10, 000–99, 999 parasites/µL), and hyperparasitaemia (≥100, 000 parasites/µL) (43, 44).

#### 2.6.2 Haemoglobin measurement and anaemia classification

Haemoglobin (Hb) concentration was measured, and each participant’s Hb value was recorded in grams per decilitre (g/dL). Anaemia was classified using haemoglobin thresholds as follows: mild anaemia, 10.0–10.9 g/dL; moderate anaemia, 7.0–9.9 g/dL; and severe anaemia, <7.0 g/dL (43). Participants with Hb values outside these ranges were classified as not anaemic. For malaria-associated physiological disturbance-related interpretation, Hb concentration was considered alongside parasite density, fever, and platelet count as an indicator of malaria-associated physiological disturbance (44, 45).

### 2.7 Measurement of plasma IL-6 and HO-1

Plasma concentrations of haem oxygenase-1 (HO-1) were measured using a Human Haem Oxygenase-1 ELISA kit (Boster Biological Technology, USA, Cat. No. EKC33968) according to the manufacturer’s instructions. The assay was performed using a 96-well microplate format designed for the quantitative detection of HO-1 in human serum and plasma samples. The kit has a detection range of 15.6–1000 ng/mL with an analytical sensitivity of 3.9 ng/mL.

Plasma interleukin-6 (IL-6) concentrations were measured using the Human IL-6 DuoSet ELISA Kit (R&D Systems, Cat. No. DY206, Minneapolis, MN, USA). Samples and standards were assayed in duplicate according to the manufacturer’s instructions.

Optical densities for both analytes were recorded at 450 nm and 630 nm, with 630 nm serving as the reference wavelength for background correction, using a BioTek ELx800 microplate reader (BioTek Instruments, USA). Concentrations were interpolated from four-parameter logistic standard curves (46).

### 2.8 Evaluation of diagnostic performance

Receiver operating characteristic (ROC) curve analysis was performed to evaluate the diagnostic performance of IL-6, HO-1, and their combined model. The area under the curve (AUC) was calculated for each biomarker. Optimal cut-off values were determined using Youden’s index, and corresponding sensitivity and specificity were reported. Diagnostic performance was assessed based on the ability of each biomarker to discriminate between malaria-positive and malaria-negative individuals, consistent with established approaches (8). To facilitate interpretation, AUC values were categorized into predefined diagnostic performance ranges, as shown in **Table 1**.

**Table 1:** Interpretation of area under the curve (AUC) values for diagnostic performance .

AUC range	Interpretation
0.90–1.00	Excellent
0.80–0.89	Very good
0.70–0.79	Good
0.60–0.69	Satisfactory
0.50–0.59	Poor/unsatisfactory

### 2.9 Statistical analysis

All statistical analyses were performed independently of laboratory procedures using Python version 3.14.2 with NumPy 2.4.1, Pandas 2.3.3, SciPy 1.17.0, Matplotlib 3.10.8, and Scikit-learn 1.8.0.

Continuous variables were non-normally distributed and are therefore summarized as medians and interquartile ranges (IQR), while categorical variables are presented as frequencies and percentages. Comparisons between microscopy-positive and microscopy-negative participants were performed using the Mann-Whitney U test for continuous variables and chi-square or Fisher exact tests for categorical variables, as appropriate. Comparisons across parasite-density categories were conducted using the Kruskal-Wallis test.

Associations between biomarkers and clinical variables were assessed using Spearman’s rank correlation coefficient (rho). Diagnostic performance was evaluated using receiver operating characteristic (ROC) analysis, with area under the curve (AUC), 95% confidence intervals, sensitivity, specificity, and optimal cutoffs defined by the Youden index (47, 48). Exploratory correlation analyses also report false-discovery-rate (FDR) adjusted q values (49, 50). All tests were two-tailed, and p< 0.05 was considered statistically significant.

## 3 Results

### 3.1 Cohort characteristics and clinical profile

The analytical cohort comprised 304 participants: 152 microscopy-positive and 152 microscopy-negative. Age and sex distributions were similar between groups, but microscopy-positive participants had substantially greater physiological disturbance. Fever was present in 68.4% of microscopy-positive participants compared with 16.4% of microscopy-negative participants, and any anaemia was present in 78.9% versus 12.5%, respectively. Summary statistics of haematological indices, fever, and anaemia status across malaria-positive and malaria-negative participants are presented in **Tables 2A, B**.

**Table 2A:** Continuous clinical and haematological variables by microscopy-defined malaria status.

Variable	Malaria-positive (n=152)	Malaria-negative (n=152)	P
Age (years)	28.00 (14.00-40.00)	27.00 (16.75-38.00)	0.918
Temperature (°C)	38.59 (36.87-39.13)	36.74 (36.51-37.12)	<0.001**
Red blood cells (×10^12/^L)	4.07 (3.74-4.42)	5.04 (4.64-5.43)	<0.001**
Haemoglobin (g/dL)	10.20 (8.30-11.71)	13.79 (12.55-14.88)	<0.001**
Haematocrit (%)	33.12 (28.55-36.37)	42.05 (39.31-44.81)	<0.001**
MCV (fL)	81.75 (77.11-89.35)	88.88 (84.79-94.06)	<0.001**
MCH (pg)	27.93 (24.73-29.90)	30.08 (28.33-31.15)	<0.001**
MCHC (g/dL)	32.18 (30.98-33.59)	33.99 (33.10-35.11)	<0.001**
White blood cells (×10^9^/L)	7.85 (6.16-9.16)	6.44 (5.33-7.62)	<0.001**
Neutrophils (%)	57.49 (52.24-64.01)	53.36 (47.63-58.68)	<0.001**
Lymphocytes (%)	32.16 (26.65-38.70)	35.62 (31.66-39.92)	<0.001**
Monocytes (%)	4.80 (3.44-6.52)	5.22 (3.33-6.52)	0.663
Eosinophils (%)	2.33 (1.44-3.14)	2.94 (2.10-3.99)	<0.001**
Platelets (×10^9^/L)	142.94 (103.65-173.38)	260.48 (222.82-308.96)	<0.001**

Values are median (IQR). P values are from Mann-Whitney U tests. All significant comparisons in this table remained significant after false-discovery-rate correction [55]. Strong significant differences are marked with **.

**Table 2B:** Categorical variables by microscopy-defined malaria status.

Variable	Malaria-positive (n=152)	Malaria-negative (n=152)	P
Male sex, n (%)	68 (44.7%)	77 (50.7%)	0.358
Fever present, n (%)	104 (68.4%)	25 (16.4%)	<0.001
Anaemia, n (%)	120 (78.9%)	19 (12.5%)	<0.001**
Anaemia severity: None, n (%)	32 (21.1%)	133 (87.5%)	<0.001**
Mild anaemia, n (%)	23 (15.1%)	18 (11.8%)	
Moderate anaemia, n (%)	68 (44.7%)	1 (0.7%)	
Severe anaemia, n (%)	29 (19.1%)	0 (0.0%)	

P values are from Fisher exact tests except anaemia-malaria-associated physiological disturbance distribution, which uses a chi-square test across categories [56]. ** p < 0.001.

**Figure 3:**
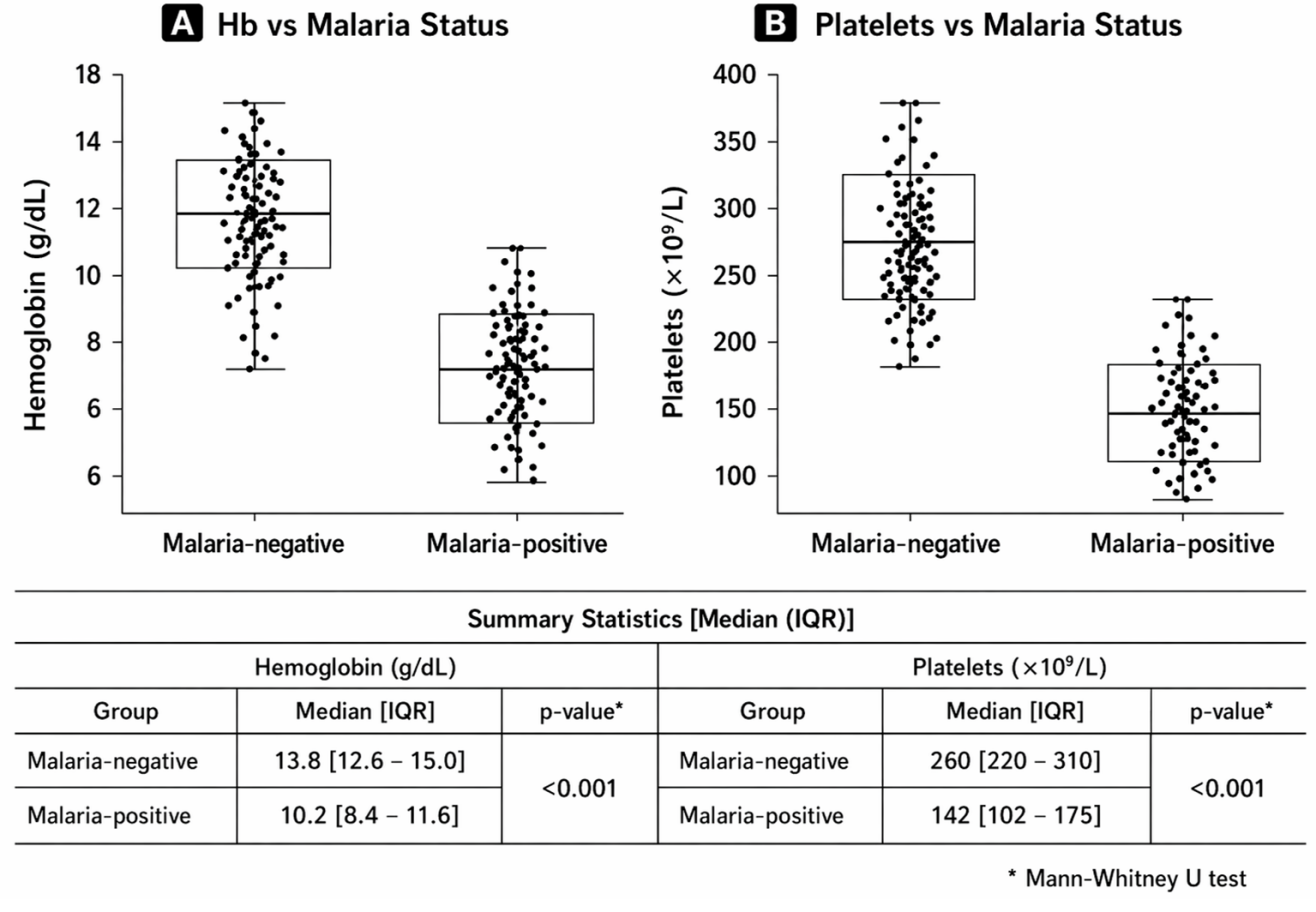
Distribution of haemoglobin **(A)** and platelet count **(B)** by microscopy-confirmed malaria status.

Microscopy-positive participants demonstrated significantly lower red blood cell count, haematocrit, mean corpuscular volume (MCV), mean corpuscular haemoglobin (MCH), and mean corpuscular haemoglobin concentration (MCHC), alongside significantly higher body temperature and white blood cell count (all p< 0.001). Marked reductions in haemoglobin and platelet counts among microscopy-positive participants are illustrated in **Figure 3**.

### 3.2 Plasma IL-6 and HO-1 levels by malaria status

The integrated biomarker subset included 40 participants (22 malaria-positive and 18 malaria-negative). Plasma IL-6 and HO-1 concentrations stratified by malaria status are presented in **Table 3**, which also includes the demographic characteristics of the biomarker subset. Plasma IL-6 levels were significantly higher in malaria-positive participants compared to malaria-negative individuals (median: 141.28 vs 106.58 pg/mL; p = 0.027). In contrast, HO-1 levels showed a higher median in malaria-positive participants, but this difference was not statistically significant (median: 230.98 vs 193.73 ng/mL; p = 0.131).

**Table 3:** Plasma biomarker concentrations and demographic characteristics by malaria status in the integrated subset.

Variable	Malaria-positive (n = 22)	Malaria-negative (n = 18)	p-value
Age (years)	29 (21–38)	22 (12–38)	0.18
Sex (Male/Female)	12/10	7/11	0.34
IL-6 (pg/mL)	141.28 (113.60–173.44)	106.58 (10.00–118.48)	0.027*
HO-1 (ng/mL)	230.98 (205.33–295.73)	193.73 (168.55–278.77)	0.131

*Statistically significant at p< 0.05. Values are presented as median (IQR). p-values for continuous variables were obtained using the Mann–Whitney U test, and categorical variables using the Chi-square test. IL-6 values were available for 39 participants because one malaria-negative sample had missing IL-6 data.

### 3.3 Variation of IL-6 and HO-1 across parasite-density categories

**Figure 4:**
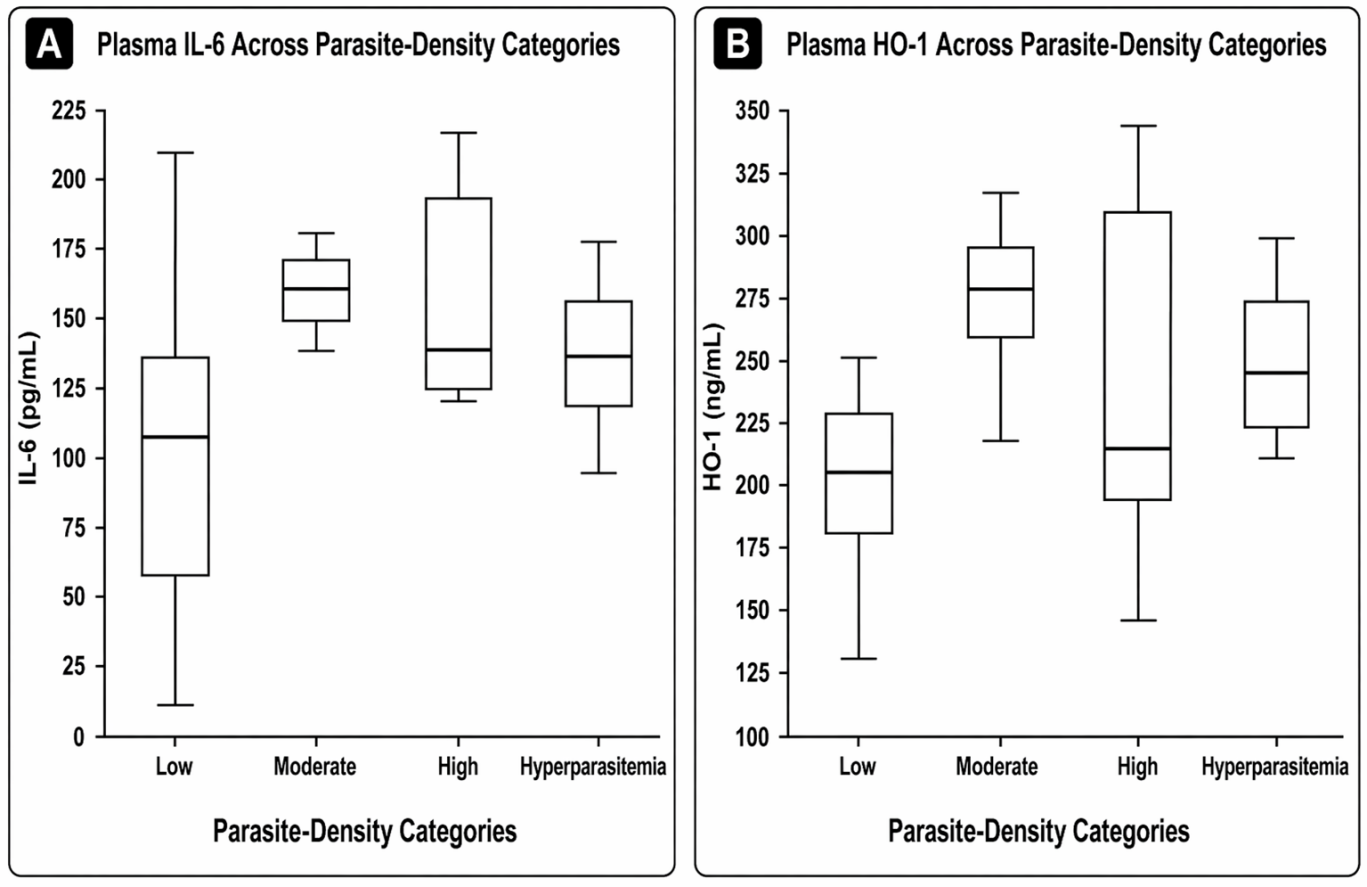
Plasma IL-6 **(A)** and HO-1 **(B)** concentrations across parasite-density categories among microscopy-positive participants.

Among the 22 microscopy-positive participants included in the biomarker subset, parasite density was categorized as low (n = 7), moderate (n = 4), high (n = 7), or hyperparasitaemia (n = 4) (44). Plasma IL-6 and HO-1 concentrations across these categories are presented in **Figure 4**. No statistically significant differences were observed across parasite-density categories for either IL-6 or HO-1.

### 3.4 Correlation analysis of biomarkers and clinical parameters

**Table 4** presents Spearman correlation coefficients between biomarkers and key clinical variables in the integrated biomarker subset. IL-6 was positively correlated with HO-1, parasitaemia, and temperature, and negatively correlated with haemoglobin. HO-1 was positively correlated with temperature and negatively correlated with haemoglobin, whereas its correlation with parasitaemia did not reach statistical significance. The strongest association was between IL-6 and HO-1 (rho = 0.702, p< 0.001). IL-6 correlated with parasitaemia (rho = 0.387, p = 0.015), temperature (rho = 0.432, p = 0.006), and lower haemoglobin (rho = -0.417, p = 0.008). HO-1 correlated with temperature (rho = 0.412, p = 0.008) and lower haemoglobin (rho = -0.367, p = 0.020).

**Table 4:** Key Spearman correlations in the integrated biomarker subset.

Biomarker	Variable	N	Spearman rho	p	FDR q
IL-6 (pg/mL)	HO-1 (ng/mL)	39	0.702	<0.001**	<0.001
	Parasitaemia (/µL)	39	0.387	0.015*	0.025
	Temperature (°C)	39	0.432	0.006*	0.017
	Haemoglobin (g/dL)	39	-0.417	0.008*	0.017
	Platelets (×10^9^/L)	39	-0.261	0.109	0.121
HO-1 (ng/mL)	Parasitaemia (/µL)	40	0.267	0.096	0.120
	Temperature (°C)	40	0.412	0.008*	0.017
	Haemoglobin (g/dL)	40	-0.367	0.020*	0.028
	Platelets (×10^9^/L)	40	-0.216	0.181	0.181

Values shown are Spearman’s rank correlation coefficients (rho). FDR q-values represent p-values adjusted for multiple comparisons using the false discovery rate approach. Associations with q< 0.05 were considered robust after correction for multiple testing. *p< 0.05; **p< 0.001.

### 3.5 Diagnostic performance of biomarkers (ROC analysis)

**Figure 5:**
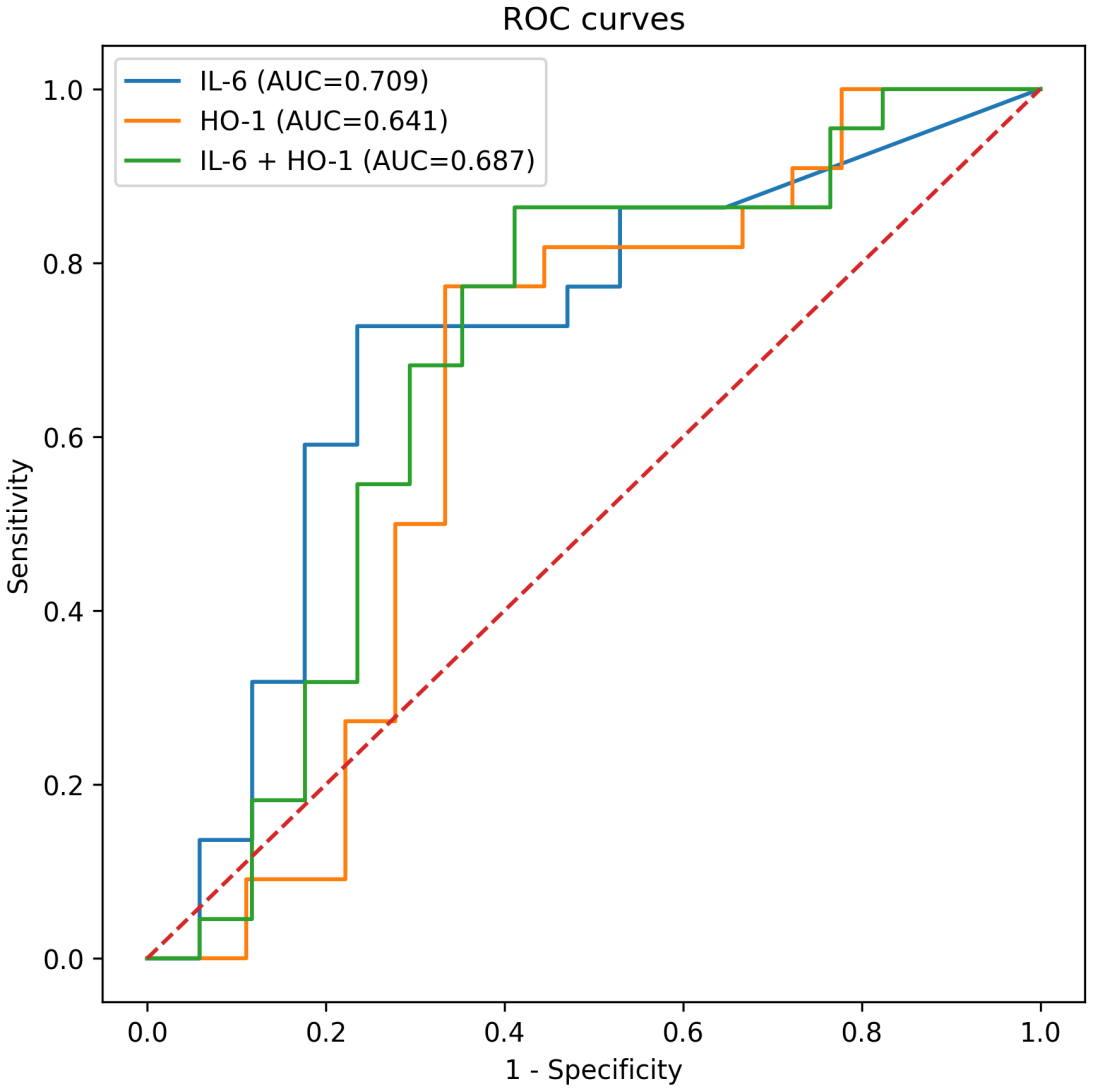
Receiver operating characteristic (ROC) curves for IL-6, HO-1, and their combined model.

Receiver operating characteristic (ROC) curve analysis was performed to evaluate the diagnostic performance of IL-6, HO-1, and their combined model in distinguishing malaria-positive from malaria-negative participants. As shown in **Figure 5**, IL-6 demonstrated the highest discriminative ability, followed by the combined IL-6 + HO-1 model, while HO-1 alone showed lower performance.

ROC curves illustrating the diagnostic performance of IL-6 (AUC = 0.709), HO-1 (AUC = 0.641), and the combined IL-6 + HO-1 model (AUC = 0.687) in discriminating malaria-positive from malaria-negative participants. The dashed red line represents the line of no discrimination (AUC = 0.5).

IL-6 demonstrated acceptable discriminatory performance, with an area under the curve (AUC) of 0.709, sensitivity of 72.7%, and specificity of 76.5%. In contrast, HO-1 showed more modest discrimination, with an AUC of 0.641, sensitivity of 77.3%, and specificity of 66.7%. The combined IL-6 + HO-1 logistic regression model yielded an AUC of 0.687 (95% CI 0.456–0.882), with improved sensitivity (86.4%) but reduced specificity (58.8%).

## 4 Discussion

This study evaluated plasma biomarkers haem oxygenase-1 (HO-1) and interleukin-6 (IL-6) as potential adjunct indicators of malaria disease burden in a hospital-based, endemic population in southwestern Nigeria.

The findings show that plasma IL-6 was significantly elevated in malaria-positive participants, whereas HO-1 showed a higher median concentration that did not reach statistical significance. This suggests that IL-6 more clearly distinguished malaria status in this cohort, while HO-1 may reflect a more variable host-response pattern. Our IL-6 findings are consistent with previous studies showing increased circulating IL-6 in malaria infection (16, 18, 51). However, in the present study, IL-6 did not differ significantly across parasite-density categories within the malaria-positive subset (**Figure 4**).

Taken together, these findings support IL-6 as a more responsive marker of malaria-associated inflammation in this dataset (17), while HO-1 may provide complementary information related to oxidative or haemolytic stress rather than serving as a strong standalone discriminator of malaria status or parasite-density category (10, 52). Kern and colleagues first highlighted the broader cytokine surge associated with complicated falciparum malaria (53), while Mshana et al. documented elevated IL-6 levels alongside other inflammatory cytokines in a holoendemic population (18). Wenisch et al. later demonstrated that IL-6 levels were particularly high in severe malaria presentations involving cerebral malaria or renal failure (19). Day et al. further showed that elevated IL-6, together with imbalances between pro- and anti-inflammatory cytokines, carried prognostic value in severe malaria (54).

More recently, Wilairatana et al. synthesised available studies in a systematic review and meta-analysis and concluded that IL-6 is significantly increased in severe compared with non-severe malaria (16), a broader inference that was reinforced by the larger inflammatory biomarker meta-analysis by Hashmi et al. (20). In that context, the positive correlation between IL-6 and parasite density observed in our cohort is biologically plausible and consistent with the concept that higher parasite biomass drives greater systemic inflammatory activation (6).

At the same time, our study contributes nuance. That is, current findings extend previous observations by providing additional evidence on the relationship between these biomarkers and malaria-associated physiological changes. While IL-6 in our integrated subset showed acceptable discrimination for malaria positivity, its AUC did not approach the level required for standalone clinical replacement of parasite-based tests. This is consistent with the broader severe-malaria biomarker literature, which increasingly recognizes that host biomarkers are complementary rather than substitutive (21). IL-6 is a non-specific inflammatory marker that may be elevated in a wide range of infectious and inflammatory conditions. Because alternative febrile comorbidities were not systematically excluded in this study, the observed IL-6 elevations should be interpreted as reflecting inflammatory activation in malaria-positive participants rather than malaria-specific responses alone (20, 25, 27). This likely explains why even statistically meaningful IL-6 differences do not necessarily translate into outstanding real-world diagnostic specificity (55).

The HO-1 findings are particularly informative when interpreted in the context of haemolysis-driven oxidative stress during malaria infection. Experimental and translational work has established a strong mechanistic basis for HO-1 involvement in malaria pathogenesis. Ferreira et al. proposed that free haem is a central driver of severe malaria pathophysiology and demonstrated that induction of HO-1 and carbon monoxide generation can be protective in experimental models (9). Subsequent work by Pereira and colleagues extended this haem/HO-1 axis to malaria-associated acute lung injury and acute respiratory distress syndrome, demonstrating increased HO-1 expression and protective effects of HO-1 induction in murine models (56, 57). Broader reviews have reinforced the concept that oxidative stress is a major determinant of host injury in malaria, and that HO-1 sits at the centre of the host’s attempt to detoxify cell-free haem (10, 15, 58).

However, human data also suggest that the role of HO-1 in malaria is complex. Walther et al. reported that higher HO-1 levels and promoter variants associated with greater inducibility were linked to severe malaria in Gambian children (22). Aubouy et al. observed that monocyte HO-1-related gene expression in pregnancy-associated malaria was associated with favourable outcomes in that context (23). These apparently divergent observations point to the context-dependent role of HO-1: a pathway that may be compensatory and protective at one stage of disease yet may also index a greater burden of haemolysis and host stress when markedly elevated in severe illness (58). Another notable finding in this study was the association of IL-6 and HO-1 with each other and with key haematological parameters. This pattern is clinically relevant because haemolysis, thrombocytopenia, fever, and inflammation often evolve together during malaria infection (42, 59, 60). Prior studies have shown that inflammatory cytokines are associated with malaria-related thrombocytopenia (61), while broader biomarker analyses have emphasised that clinically useful risk stratification may require multi-marker panels rather than single analytes (6, 21, 27). Our results support that direction. Even though the combined IL-6 + HO-1 model did not dramatically outperform IL-6 alone in the integrated complete-case analysis, the joint evaluation remains valuable because it captures two biologically distinct, clinically relevant dimensions of the host response.

In comparison with more recent biomarker studies, the current findings situate the novelty of this work, which brings additional perspective by examining both inflammatory (IL-6) and oxidative stress (HO-1) pathways within the same host-response framework. Nahm (62) emphasised that ROC analysis is useful for comparing diagnostic performance and selecting cut-off values, but that AUC should be interpreted carefully within the clinical context rather than overstated as proof of strong diagnostic utility. Çorbacıoğlu and Aksel (63) further noted that modest AUC values indicate limited discrimination, supporting cautious interpretation when biomarker performance does not clearly separate groups. Consistent with this, IL-6 showed better discriminatory performance than HO-1, whereas HO-1 demonstrated only limited discrimination and no significant difference between groups. The combined model did not meaningfully improve performance beyond IL-6 alone (**Figure 5**).

Contemporary studies have explored endothelial or multi-protein signatures in severe paediatric malaria or malaria in pregnancy. Kanoi et al. identified HO-1 among differentially expressed plasma proteins in pregnant women with falciparum malaria using high-throughput proteomics (24), while Varo et al. recently demonstrated that host biomarkers and parasite biomass can support risk stratification in severe paediatric malaria (6). Yet these studies were conducted in specialised populations and often relied on proteomic or multiplex discovery platforms rather than simple ELISA-based quantification. By contrast, the present study examined both HO-1 and IL-6 in a general malaria-exposed clinical population and evaluated their joint ROC performance using standard plasma ELISA assays. That translational simplicity is a practical strength, because any biomarker intended for wider use in endemic settings must ultimately be measurable using accessible laboratory methods (64).

An important methodological consideration in this study relates to how malaria-associated physiological disturbance was conceptualised. Because the cohort was not assembled as a classical severe-malaria case series, formal classification using WHO-defined severe malaria criteria was not feasible. Instead, malaria-associated physiological disturbance was inferred from parasite density, interpreted according to WHO-recommended thresholds (39, 43, 44), along with haematological and clinical indicators, including haemoglobin concentration, platelet count, and fever. This approach is particularly relevant for studies conducted in endemic populations where patients often present across a broad clinical spectrum rather than within clearly defined severe and non-severe categories (65). In such contexts, malaria-associated physiological disturbance-related indicators may provide more informative insight into host physiological stress than binary clinical classifications. For this reason, the present study deliberately adopts the phrasing “associated with malaria or malaria-associated physiological disturbance” rather than implying direct validation against WHO syndromic endpoints. This conservative framing improves clinical interpretability and aligns with emerging perspectives that host-response biomarkers should be viewed as adjunct indicators of disease burden, rather than replacements for established clinical malaria-associated physiological disturbance definitions (21, 66). To reduce the risk of false-positive findings arising from multiple comparisons, false discovery rate (FDR) correction was applied to the correlation analyses. The resulting q-values represent p-values adjusted using the false discovery rate approach (**Table 4**). Associations with q< 0.05 were considered robust after correction for multiple testing, whereas those with higher q-values were interpreted more cautiously (49). Given the exploratory nature of the biomarker subset, formal power calculations were limited; however, the sample size was sufficient to support exploratory correlation and ROC analyses, while findings should be interpreted cautiously and validated in larger cohorts. Overall, our findings reinforce the perspective that malaria pathogenesis cannot be understood solely through the lens of parasite biology and vector transmission. Host physiological responses appear to play a major role in determining clinical outcomes, as individuals with similar parasite burdens frequently experience markedly different disease manifestations. Our observations highlight the importance of host oxidative stress and inflammatory pathways as complementary dimensions of malaria pathophysiology that warrant greater consideration alongside conventional parasitological assessments. In resource-constrained endemic settings, plasma IL-6 and HO-1 are unlikely to replace microscopy or RDTs, but they may help refine biological stratification of patients, identify those with more pronounced host stress, and guide future multi-marker malaria-associated physiological disturbance panels that combine parasite and host-response information.

## 5 Study limitations

This study has several limitations. First, the cross-sectional design does not permit temporal inference regarding biomarker dynamics during malaria infection. Second, IL-6 is a non-specific inflammatory marker, and alternative febrile comorbidities were not systematically excluded (67). Third, the integrated biomarker subset was relatively small and should be regarded as exploratory. Accordingly, these findings should be interpreted cautiously and validated in larger prospective cohorts. Future studies should include larger prospective cohorts, explicit WHO severe-malaria phenotyping, and comparison with other host-response candidates such as angiopoietins, C-reactive protein (CRP), and endothelial activation markers (6, 21, 27, 66).

## 6 Conclusion

Plasma IL-6 was significantly associated with malaria positivity, whereas HO-1 showed a non-significant upward trend and limited discriminatory ability. These findings support IL-6 as a clearer inflammatory marker in this cohort, while HO-1 may reflect oxidative or haemolytic host stress but should not be interpreted as a strong standalone discriminator based on the present data. Larger studies are needed to clarify the clinical relevance of HO-1 and the potential added value of combined host biomarkers. These findings support IL-6 as a clearer inflammatory marker in this cohort and may provide additional biological insight beyond parasite detection and conventional haematological indices in understanding malaria-associated physiological disturbance in endemic settings. Larger prospective studies are needed to validate these observations and clarify their clinical relevance.

## Data Availability

The raw data supporting the conclusions of this article will be made available by the authors, without undue reservation.
